# Rhinology, Laryngology and Otology, and Their Significance in General Medicine

**Published:** 1900-12

**Authors:** 


RHINOLOGY, LARYNGOLOGY AND OTOLOGY, AND THEIR SIGNIFICANCE IN GENERAL MEDICINE. By E. P. Friedrich, M.D., Leipzig. Edited by H. Holbrook Curtis, M.D., New York. Published by W. B. Saunders & Co., Philadelphia. Price, $2.50 net.

Several years ago an English translation was made of Prof· Knies' work on "The Eye in General Diseases," which proved to be, and still is, a most valuable contribution to the library of every ophthalmologist. The above work by Dr. Friedrich is likewise a valuable contribution to medical literature, and as the subject-name indicates, will be of most value to those practicing otology, rhinology and laryngology. Just such a work has been needed for some time, for, although the various subjects herein discussed could be found in a fragmentary way in every good text-book on these subjects, yet there was needed a work which would group the various subjects in a manner which could be utilized for ready reference. There are no illustrations in the book, and every page is given up to valuable reading-matter. In this day of quickly made specialists who, perhaps, have never had any general practice nor served in a general hospital, such a work will prove to them of inestimable value. Every specialist in these organs treated should possess this book and make use of it by daily reading, for such are very prone to limit all pathologic conditions to the organs which they treat and forget the relationship existing between all parts of the human anatomy. The work is well translated and put into good Engish, and we predict for it a ready demand.

